# GUIDANCE2: accurate detection of unreliable alignment regions accounting for the uncertainty of multiple parameters

**DOI:** 10.1093/nar/gkv318

**Published:** 2015-04-16

**Authors:** Itamar Sela, Haim Ashkenazy, Kazutaka Katoh, Tal Pupko

**Affiliations:** 1 Department of Cell Research and Immunology, George S. Wise Faculty of Life Sciences, Tel-Aviv University, Tel-Aviv 6997801, Israel; 2 Immunology Frontier Research Center, Osaka University, Suita, Osaka 565-0871, Japan; 3 Computational Biology Research Center, The National Institute of Advanced Industrial Science and Technology (AIST), Tokyo 135-0064, Japan

## Abstract

Inference of multiple sequence alignments (MSAs) is a critical part of phylogenetic and comparative genomics studies. However, from the same set of sequences different MSAs are often inferred, depending on the methodologies used and the assumed parameters. Much effort has recently been devoted to improving the ability to identify unreliable alignment regions. Detecting such unreliable regions was previously shown to be important for downstream analyses relying on MSAs, such as the detection of positive selection. Here we developed GUIDANCE2, a new integrative methodology that accounts for: (i) uncertainty in the process of indel formation, (ii) uncertainty in the assumed guide tree and (iii) co-optimal solutions in the pairwise alignments, used as building blocks in progressive alignment algorithms. We compared GUIDANCE2 with seven methodologies to detect unreliable MSA regions using extensive simulations and empirical benchmarks. We show that GUIDANCE2 outperforms all previously developed methodologies. Furthermore, GUIDANCE2 also provides a set of alternative MSAs which can be useful for downstream analyses. The novel algorithm is implemented as a web-server, available at: http://guidance.tau.ac.il.

## INTRODUCTION

Multiple sequence alignment (MSA) is a key component in almost every comparative analysis of biological sequences (DNA or proteins). Moreover, MSA reconstruction is often the first step in bioinformatic pipelines, where MSA is later used for further analyses. Over the years, many algorithms and approaches aiming at constructing such alignments have been developed, showing a steady improvement in the accuracy of the resulting MSA (1–10). However, studies that aimed to objectively evaluate the accuracy of several MSA algorithms have shown that even the most accurate alignment algorithms available today are still subject to a substantial amount of errors (11–13).

Alignment inference is a complicated statistical estimation problem, in which alignment uncertainty originates from both the stochastic nature of the evolutionary process and computational limitations of current evolutionary models and alignment methodologies. The substantial uncertainty when inferring optimal MSAs is manifested by the large differences in the resulting alignments among existing alignment algorithms (14). Thus, it appears that not any inferred alignment should be used as granted for downstream analyses in a bioinformatic pipeline, as any specific MSA is likely to contain wrongly aligned regions. Indeed, errors in the MSA may bias downstream analyses, such as the detection of positive selection (15,16), and likelihood-based tests for comparing phylogenetic tree topologies (17).

Several methods aimed at estimating unreliable alignment regions were previously developed (18–31). Among these methodologies, ZORRO (29) and PSAR (27) use hidden Markov models to detect uncertainty in pairwise alignments, which are the building blocks of the MSA in progressive alignment algorithms. Unreliable alignment regions are often associated with high sequence variability, both in terms of the number of amino-acid replacements and in the number and lengths of indels (gaps). Several methodologies utilize this association to detect unreliable alignment regions. For example, trimAl and ALISCORE consider regions with low sequence identity and similarity as unreliable (22–24). Gblocks scores as reliable only blocks in the alignment that have a low number of gaps (18). The Noisy algorithm associates unreliability with regions suspected as homoplasious positions (20). Finally, the TCS methodology uses a library of pairwise alignments to score positions in the evaluated MSA (31).

Another class of alignment reliability methods is based on a consistency principle: alignment regions that are shared among a large number of alternative MSAs built from the same sequence data are considered to be more reliable. Such consistency-based approaches differ in the way these alternative MSAs are generated. The heads or tails (HoT) methodology (19,21) generates alternative alignments by utilizing the fact that when aligning a pair of sequences, often more than one optimal solution exists. HoT specifically detects two extreme co-optimal solutions for each pair of sequences aligned by a progressive alignment approach. This is achieved by aligning the two sequences twice: once in their original order of characters (‘the head’) and once with the characters in reverse order (‘the tail’). HoT then combinatorially propagates the uncertainty when joining sequences or partial alignments to the growing MSA, thus generating a large set of alternative MSAs. The GUIDANCE algorithm (25,26) generates alternative MSAs by utilizing the observation that alignments substantially vary when given alternative tree topologies to guide the progressive alignment. Specifically, GUIDANCE first constructs a large number of alternative tree topologies by bootstrapping the MSA generated by the alignment program. Each such bootstrap tree is next used as a guide tree to re-align the original sequences. The number of alternative alignments is thus dictated by the number of alternative trees and, in theory, some of these alignments can appear more than once.

Many of the above described methods were only recently developed and most were shown to outperform Gblocks, the classic and most commonly used alignment reliability methodology. In this study, we aimed to systematically compare seven of the more recent algorithms to detect unreliable regions, GUIDANCE (26), HoT (21), ALISCORE (24), trimAl (22), TCS (31), ZORRO (29) and Noisy (20), on a wide range of both simulated and structure-based alignments. Following the comparison among the different methodologies, we realized the importance of modeling uncertainty in the propensity to open gap characters (32) as well as the uncertainty of the guide tree and co-optimal pairwise alignment solutions. We integrated all these insights into a new version of the GUIDANCE algorithm and showed that the new integrated version (GUIDANCE2) outperforms all previously developed methods. Furthermore, GUIDANCE2 produces a set of alternative alignments, which can be valuable in downstream analyses.

## MATERIALS AND METHODS

### Data sets

The performance of various MSA reliability assessment methodologies was tested on empirical benchmarks as well as on simulated data, which differ in various parameters such as number and length of sequences and average sequence identity (Supplementary Table S1). Two empirical benchmark data sets were used: BAliBASE 3 (33) and HOMSTRAD (34). For HOMSTRAD we used the 232 MSAs with more than three sequences. For BAliBASE we used reference sets 1–5 and evaluations were performed using core blocks columns only. Core blocks are defined for the true alignments. For assessment, not only core block residues are aligned and hence, when comparing the inferred alignment with the true alignment, one has to define which columns in the inferred alignment correspond to core blocks. Here, columns in the inferred alignment were compared to core block columns only if they contained two or more residues that belong to the core blocks in the true alignment.

Sequences were simulated using INDELible (35). In order to have realistic parameters for the simulations, we first selected 541 MSAs from the OrthoMaM (version 8) database (36), for which coding sequences (CDS) are available for all 40 mammals included in the database. Each such MSA is associated with a tree (and branch lengths), shape parameter of the gamma distribution (alpha) and proportion of invariant positions (pinv). We simulated 541 MSAs based on these parameters, with amino-acid replacements following the LG matrix (37). INDELible parameter for max indel length was set to be the minimum between 10% of the alignment length and 25. The length of the root sequence was arbitrarily chosen to be 66% of the OrthoMaM alignment length. All other parameters were set to the default. This parameter setup resulted in MSAs similar to OrthoMaM alignments (based on visual comparison of the alignments’ total length, number and length of indels). Following, we refer to this data set as ‘OrthoMaM simulations’.

We used two additional simulated data sets previously generated to evaluate MSA reliability methods. Specifically, we simulated sequences using ROSE (38), according to the simulation scheme provided in the ZORRO paper (29). The second data set was part of the data set used in the trimAl study (22). We used asymmetric trees with 32 and 64 species and divergence of x0.5, x1 and x2. Using these trimAl data sets it was previously shown that MSA uncertainty impacts phylogeny inference (31).

### Alignment programs

MAFFT version 7.123b (39), PRANK version 140110 (7), ClustalW version 2.0.10 (6) and T-Coffee version 10.00 (40) were used as alignment algorithms. MAFFT, ClustalW and T-Coffee were used with default parameters. PRANK was used with the +F argument for higher accuracy of indel placement. T-Coffee, PRANK, MAFFT and ClustalW were used to show that all alignment methods have high error rates on both simulated and empirical data sets. MAFFT was used for all subsequent analyses as it is both computationally efficient and has relatively high performance on both simulated and empirical data sets. ClustalW was also used when comparing the performance of the various alignment reliability methods.

### Reliability methods

Eight reliability evaluation methods were tested. ALISCORE version 2.0, TCS and ZORRO were applied with default parameters. Noisy release 1.5.12 was run with option ‘–seqtype P’ to indicate protein sequences. trimAl version 1.2 was run with options ‘-sgc’ and ‘-scc’ to print gap percentage count for columns in the input alignment and conservation values for columns in the input alignment, respectively. trimAl, by default, outputs filtered MSA. However, here all MSA reliability algorithms were compared by testing the agreement between the reliability score of each position and whether this position is ‘true’ or not. trimAl decides whether or not to filter a position based on a gap percentage count or based on a conservation value. When we compared trimAl to other methods we used the conservation values rather than the gap percentage count as the trimAl's reliability score for each position, as it resulted in more accurate inference. GUIDANCE version 1.5; HoT version 1.6 and GUIDANCE2 were run with default parameters. Out of the possible scores calculated by TCS, GUIDANCE, HoT and GUIDANCE2, the performance was evaluated using the column score (CS).

### Evaluation methods

The alignment quality was measured at the column level to allow comparison of all reliability methodologies. To this end, each column in the inferred alignment was labeled as correctly aligned when it matched a column in the true MSA; all other cases were labeled as incorrectly aligned (this corresponds to a CS of 1 and 0, respectively). Notably, the matching between columns of any two alignments (e.g. true and inferred MSAs) was computed by representing each MSA by a C matrix (the supplementary information of (41)). Each reliability method provides a score for each column in the MSA reflecting its predicted reliability. In order to assign correct/incorrect labels to each column according to the reliability score, it is necessary to define a threshold: columns with scores above the threshold are predicted to be correctly aligned and *vice versa*. It is therefore common to quantify a predictor quality by considering the true positive rate and false positive rate over all thresholds. This information is given in the receiver operating characteristic (ROC) curves (42). ROC and the area under the curve (AUC-ROC) were calculated using ROCR (43). The area under the precision-recall curve (AUC-PR) was calculated using a java package (44).

### Algorithm

To further improve the GUIDANCE methodology, we carefully inspected erroneous columns in the MSA that were ranked as reliable when using GUIDANCE. The characterization revealed that many errors occurred in regions containing long stretches of gaps in a substantial number of sequences in the MSA. This observation suggested that considering alternative alignments generated by varying the gap opening penalty in the alignment program can be beneficial for estimating the MSA reliability. We hypothesized that it will be beneficial to combine the three following sources of uncertainty: co-optimal solutions (as used to generate perturbed MSAs in HoT); guide tree instability (GUIDANCE); and opening gap penalty (as described above) and thus we incorporated these three components in GUIDANCE2. Specifically, given a set of sequences, a reference MSA is built using default or user-defined parameters. Next, a set of alternative alignments is created by inducing perturbations using all the above three components. Uncertainty in the guide tree is generated by computing 100 bootstrap trees (45) using NJ (46) as described in the original GUIDANCE method (26). For each tree, a gap opening penalty is sampled from a uniform distribution between 1–3 for MAFFT and 4–16 for ClustalW. Finally, four co-optimal solutions were sampled for each guide-tree and gap opening combination, using the HoT methodology (21). As in GUIDANCE, given a set of perturbed alignments (the default in GUIDANCE2 is 400) we computed a reliability score for each column, residue pairs, residue and sequence in the reference alignment. For example, the CS is the frequency of the column among the perturbed alignments (see (26)).

## RESULTS

### Alignment methods are prone to error

It was previously shown that alignment programs often err (12). Here, we compared the accuracy of MAFFT, PRANK, ClustalW and T-Coffee on the BAliBASE benchmark (33) and on simulated sequences. For MAFFT, PRANK, ClustalW and T-Coffee, the average CS was 0.43, 0.34, 0.34 and 0.52, respectively, for the empirical data, and 0.56, 0.64, 0.30 and 0.48, respectively, for the simulated data set (Table 1). These results suggest that less than 45 and 65% of the columns in difficult alignment problems for the empirical data and for the simulated data, respectively, are correctly aligned. This high error rate demonstrates the high uncertainty associated with MSA inference. This together with previous results showing that generated MSAs substantially differ among MSA methodologies motivated us to quantify this uncertainty for each column and for each pair of residues, so that poorly aligned regions can be identified and accounted for in downstream analyses.

**Table 1.** Alignment performance on BAliBASE and simulated data sets as measured by CS, for MAFFT, PRANK, ClustalW and T-Coffee

	BAliBASE	OrthoMaM simulations
MAFFT	0.43 ± 0.29	0.56 ± 0.16
PRANK	0.34 ± 0.27	0.64 ± 0.15
ClustalW	0.34 ± 0.32	0.30 ± 0.15
T-Coffee	0.52 ± 0.32	0.48 ± 0.17

Scores are calculated by averaging over all MSAs; standard deviations are also indicated.

### Comparing currently available MSA reliability methods

**Figure 1.**
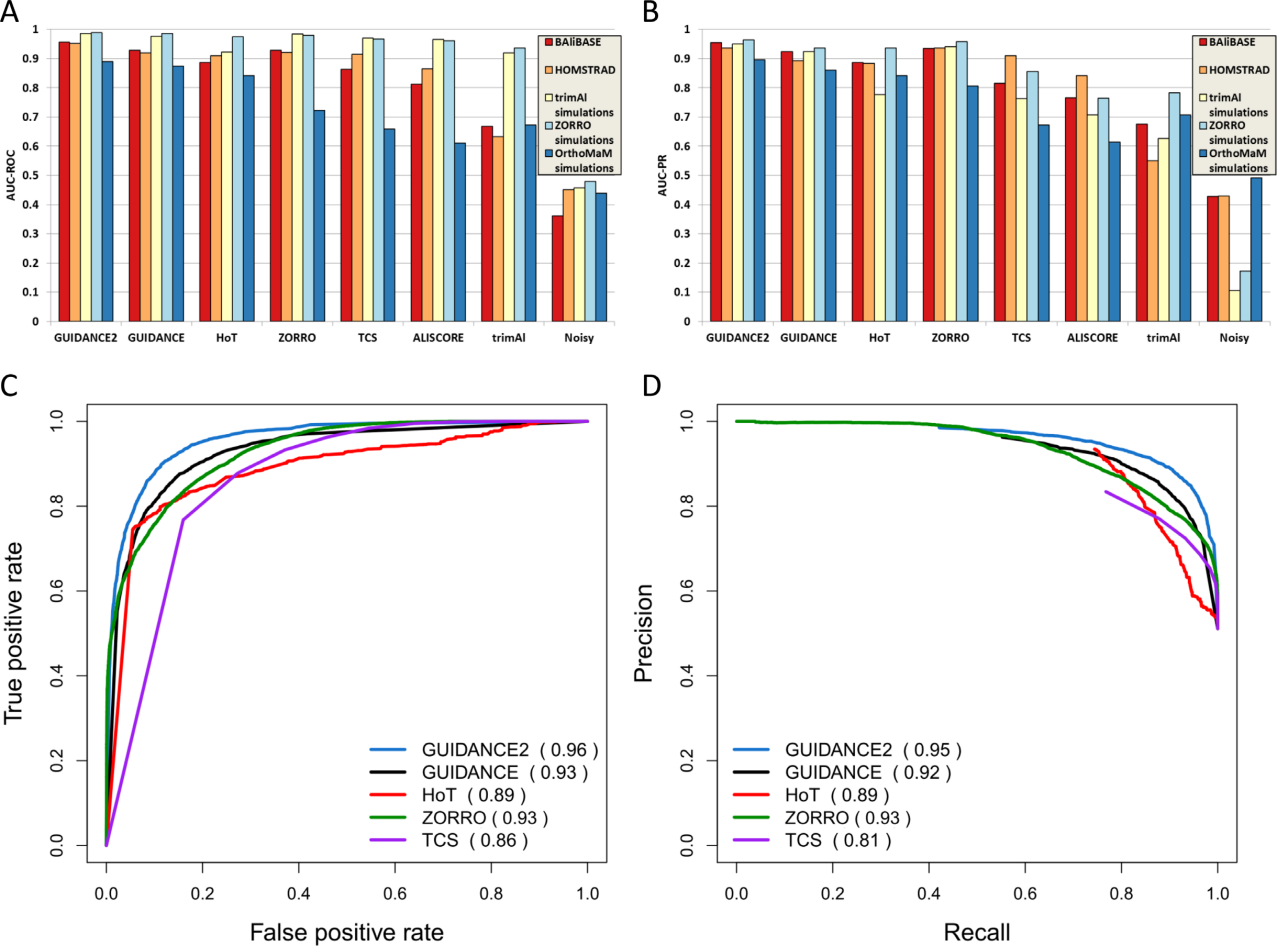
Quantitative comparison of all MSA reliability algorithms for different data sets. (**A**) AUC-ROC and (**B**) AUC-PR. Performance curves of the five leading methodologies over the BAliBASE data set. (**C**) ROC and (**D**) precision–recall.

We systematically compared the performance of various MSA reliability methods: GUIDANCE2, GUIDANCE, HoT, ALISCORE, trimAl, Noisy, TCS and ZORRO both on empirical and simulated protein sequences. As can be seen in Figure 1, GUIDANCE2 shows the best performance on both simulated and empirical benchmarks. Similar results were obtained when using ClustalW as alignment algorithm (Supplementary Figure S1). The high AUC-ROC and AUC-PR scores suggest that current methods can accurately detect erroneously aligned columns. For example, allowing a false positive rate of 0.2 (methodologies erroneously classify 20% of the incorrectly aligned columns as reliable) all the five leading methodologies considered obtain a true positive rate above 0.8 (the methods identify more than 80% of the correctly aligned columns as reliable). Labeling each column as ‘true’ or ‘false’ according to their CS, as done for the AUC-ROC and AUC-PR analyses, quantifies the ability of each reliability method to predict the accuracy of entire columns. This measure is very strict—a column is either perfectly aligned or it is labeled as ‘false’. We thus also considered the sum-of-pairs CS that quantifies the fraction of correctly aligned pairs in each column. Specifically, we calculated the Pearson correlation coefficient between each method's reliability score and the fraction of correctly aligned pairs in each column (Supplementary Figure S2). For GUIDANCE2, GUIDANCE and HoT we used the SPC score. GUIDANCE2 showed the highest Pearson correlation coefficient for the five tested data sets: BAliBASE 0.85, HOMSTARD 0.81, OrthoMaM simulations 0.87, trimAl simulated data set 0.90 and ZORRO simulated data set 0.85.

### Insights into the advantages of GUIDANCE2 over ZORRO

**Figure 2.**
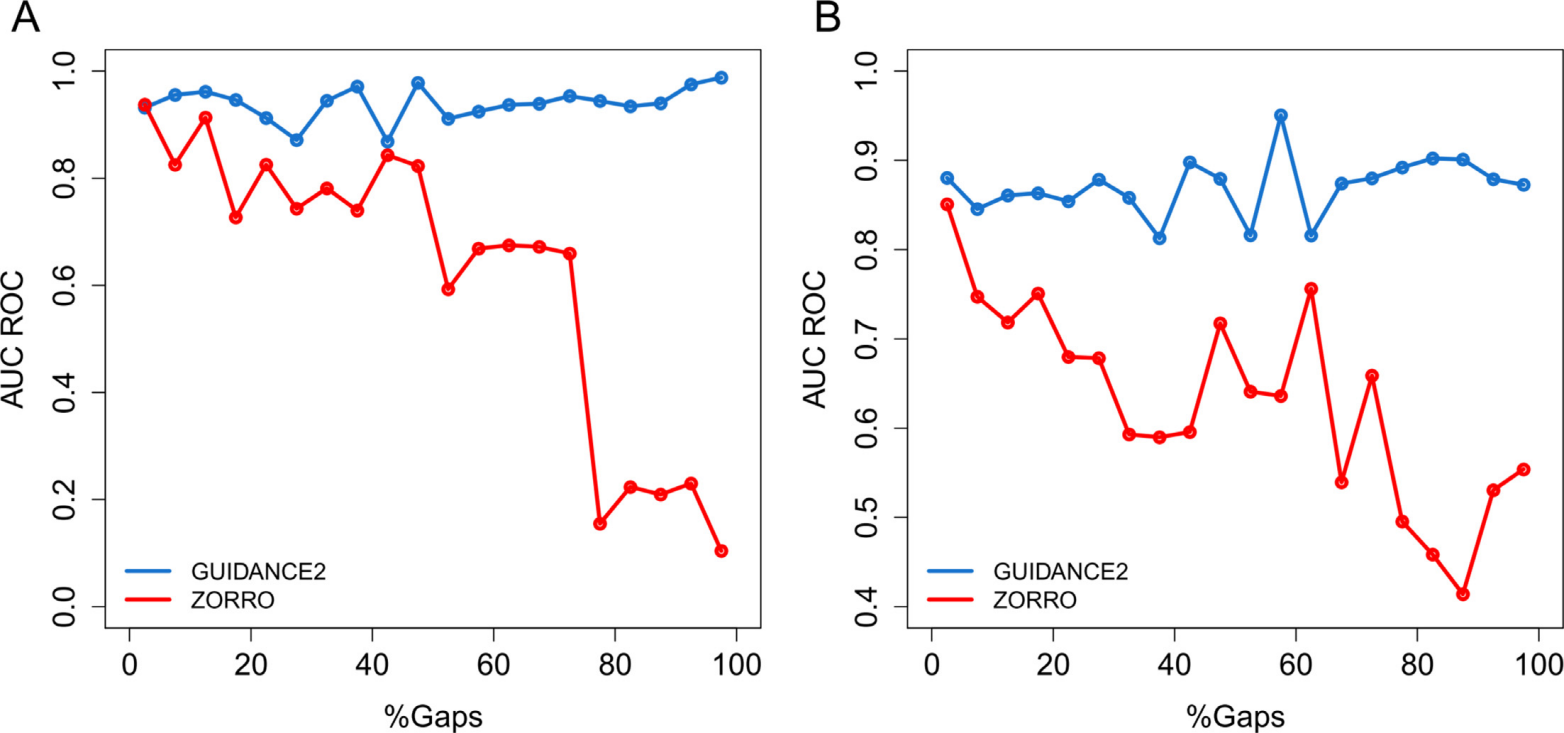
AUC ROC for columns as a function of gap percentage. (**A**) HOMSTRAD and (**B**) OrthoMaM simulations. MSAs were aligned using MAFFT.

It seems that for ZORRO, TCS and ALISCORE the performance drops for the OrthoMaM simulated data set, when measured in terms of AUC-ROC. Carefully observing wrong positions that were scored as reliable showed that such positions are usually ‘gappy’ positions. We thus tested the performance of ZORRO, the best method among those showing poor results on OrthoMaM simulations, and of GUIDANCE2 on positions with various gap percentages. Our results clearly show that on positions with extensive gappiness the performance of ZORRO is very poor (Figure 2), suggesting that ZORRO tends to erroneously favor over-aligned regions (7). This result also explains why the performance of ZORRO drops so significantly in OrthoMaM simulations compared to the performance on HOMSTRAD: the empirical benchmark contains relatively fewer positions with over 75% gaps compared with those obtained in OrthoMaM simulations (42% such positions in OrthoMaM simulations versus only 14% in HOMSTRAD).

### The contribution of the various components to the performance of GUIDANCE2

**Figure 3.**
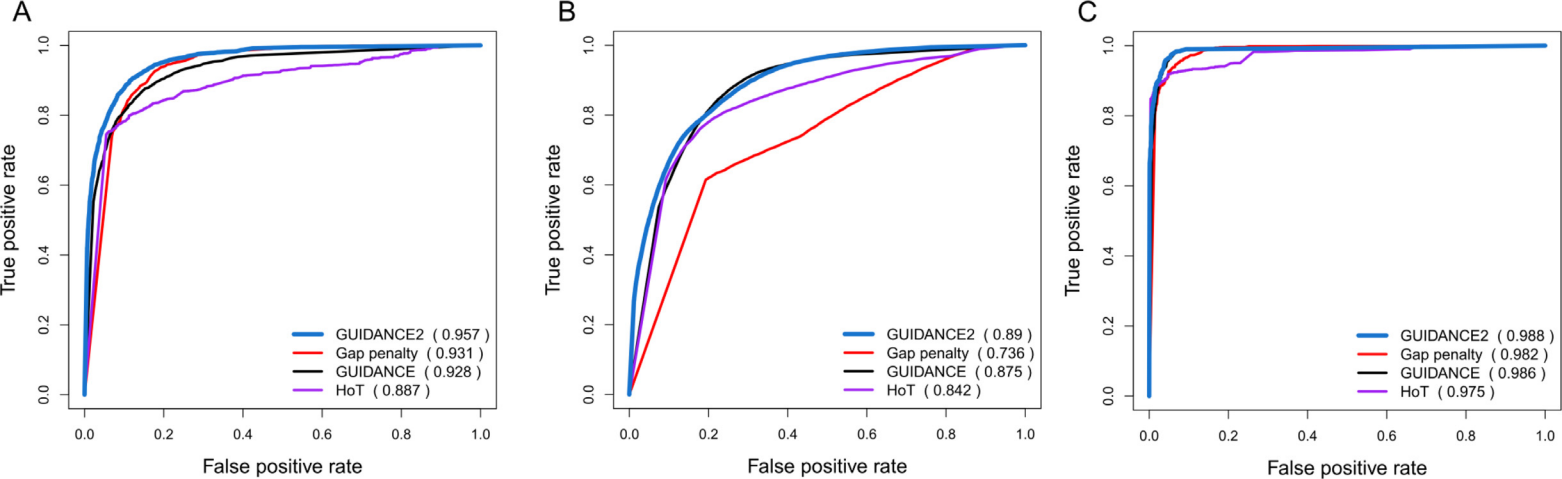
ROC curve for the performance of each GUIDANCE2 component (gap opening penalty variation is denoted as gap penalty) in detecting unreliably aligned regions for (**A**) BAliBASE, (**B**) OrthoMaM simulations and (**C**) simulations of the ZORRO paper (the ZORRO simulated data set) are shown. AUC-ROC for each component is indicated in parentheses.

As an improved combined method, GUIDANCE2 outperforms all its components when considered separately on MAFFT alignments (Figure 3): relaying on uncertainty in the guide trees (GUIDANCE), uncertainty over alternative co-optimal solutions (HoT) or uncertainty in gap penalty values. This analysis suggests that in order to generate alternative MSAs for the purpose of detecting unreliable alignment regions, for the BAliBASE data the most important factor is the gap opening score, followed by the guide tree, and the HoT component (sampling alternative MSAs with the highest score) is the least important factor (Figure 3A). For the OrthoMaM simulations, the most important factor was the guide tree uncertainty, the second was the HoT component, and the gap opening score contributed the least (Figure 3B). The contribution of each component was calculated also for the ZORRO-simulated data set. Here, the guide-tree uncertainty component (GUIDANCE) contributed the most, followed by the gap opening score, and the least was contributed by the HoT component (Figure 3C). This demonstrates that the contributions of the different components vary among data sets, further suggesting the need to integrate them within a single methodology. Notably, the gap extension score was insignificant in its contribution to the generation of alternative MSAs and was thus not included in GUIDANCE2 (data not shown).

### GUIDANCE2 as a tool to generate alternative MSAs

GUIDANCE2 (similar to GUIDANCE and HoT) is not merely a filtering methodology, but rather, it allows obtaining a set of alternative MSAs for the analyzed sequences. Such alternative MSAs can be utilized to account for MSA uncertainty in downstream analyses. We first tested whether alternative MSAs generated by GUIDANCE2 have comparable average CS, as compared to the base MSA produced by the alignment program. For each alternative MSA we computed the difference in average CS with the base MSA, denoted as ΔCS. The distributions of these values over all BAliBASE MSAs and OrthoMaM simulated data are shown in Supplementary Figure S3. Notably, 40% of alternative MSAs in BAliBASE and 25% of alternative MSAs in the simulated data set showed higher average CS than the corresponding base MSAs. This result suggests that GUIDANCE2 produces biologically reasonable alternative MSAs.

### Web-server

To enhance the usability of the suggested GUIDANCE2 methodology, we added its implementation to the GUIDANCE web-server http://guidance.tau.ac.il. GUIDANCE is a popular user friendly web-server, in which the user can reconstruct an MSA for proteins, nucleic acids or coding sequences (25). Specifically, the user can select between MAFFT, PRANK and ClustalW as the MSA construction methodology and employ GUIDANCE2 to assess the resulting alignment reliability and to generate alternative MSAs. The resulting alignment is color coded according to its reliability, thus allowing easy identification of unreliably aligned regions. Further, the user can easily mask or remove unreliably aligned regions (i.e. residue-specific, columns or sequences) from the alignment. The filtered alignment can be used for downstream analyses. The web-server includes the simulated data sets used for the performance evaluation presented in this paper, information concerning the running time on typical data sets and the stand-alone version of GUIDANCE2. We note that GUIDANCE2 is highly parallelizable and thus a significant reduction in running times is possible using the stand-alone version, which supports parallel computing.

Conveniently, MAFFT web-server http://mafft.cbrc.jp/alignment/server/ users are offered the option to evaluate the quality of the resulting MAFFT MSA with GUIDANCE2 by a direct interface between the web-servers.

## DISCUSSION

Ideally, phylogenetic trees and alignments should be co-estimated. Within the maximum-likelihood framework, this is feasible using programs such as SATe (47,48). However, this methodology does not provide reliability scores. Recent advances within the Bayesian framework allow integrating over alignments when inferring trees (9,10,49). As part of these methodologies alternative MSAs are computed, from which MSA reliability scores can easily be obtained. However, Bayesian methodologies are currently limited to very small data sets due to computational limitations. GUIDANCE2, developed here, can be viewed as a rough proxy to sampling alignments from the posterior distribution. Alternative MSAs generated by GUIDANCE2 account for uncertainty in the guide tree, in the gap opening probability and the choice among equally likely solutions. More theoretical work is needed to characterize how to generate alternative MSAs that adequately represent the MSA space. However, at least for the task of quantifying the reliability of alignment columns, we show here that using the sampled set obtained by GUIDANCE2, we can reach very high accuracy of detection: an AUC-ROC of 0.89 for OrthoMaM simulations and 0.96 for BAliBASE.

GUIDANCE2 relies on previous observations and computation achievements. For example, we show that at least when performance is measured based on the BAliBASE benchmark, considering uncertainty in the gap opening probability is a major factor contributing to GUIDANCE2 accuracy. This concept was previously suggested in the SOAP methodology (32). Unfortunately, SOAP was implemented only for ClustalW alignments. Currently, GUIDANCE2 relies on the HoT methodology to generate alternative top scoring MSAs. GUIDANCE2 could be further improved if not only top scoring alignments are considered, but instead, high scoring sub-optimal alignments are considered as well. Although theory on how to generate these sub-optimal alignments exists (50), current alignment methodologies (such as MAFFT, PRANK and ClustalW) do not provide an option to generate them as output.

A few approaches exist for taking into account uncertainty in MSAs for downstream analyses: (i) relaying on a single best alignment, (ii) relaying on a single best alignment after filtering, (iii) accounting for reliability by column weighting and (iv) weighting over multiple alternative alignments. Specifically, GUIDANCE2 provides all these options, including a set of alternative MSAs which can be utilized to account for MSA uncertainty in downstream analyses. Which is best depends on the specific application and even for a specific application, debates exist, e.g. whether or not to filter alignment columns prior to tree search (29,51,52).

The MSAs reliability methodologies were tested on both empirical and simulated data. There are conceptual differences between simulated and structural based data sets (53). While the simulation data are absolutely reliable with respect to the true MSA, it can be argued that they may not fully capture the biological complexity involved in evolutionary processes. In contrast, aligned positions based on structural data sets, such as BAliBASE, may not reflect true homology in the evolutionary sense (54). Furthermore, the empirical data sets are biased toward conserved regions among highly diverged sequences because only core blocks are taken into account (other regions are usually disregarded for benchmarking as they are considered unreliable). This bias is also reflected in the higher number of columns that are mostly gaps in the simulated data sets compared with the empirical data sets. Here, we found that different MSA reliability methodologies differ substantially in their performance on these two types of data sets because of their different gap distribution. Finally, we showed that the contribution of different factors to the accuracy of GUIDANCE2 also differs between simulation and benchmark data sets (Figure 3). This result highlights the importance of extensive benchmarking on both types of data. In the future it might be possible to generate a third type of benchmark alignments, using experimental evolution approaches (55–57).

## SUPPLEMENTARY DATA

Supplementary Data are available at NAR Online.

SUPPLEMENTARY DATA
