# Supplementary material for: GUIDANCE2: accurate detection of unreliable alignment regions accounting for the uncertainty of multiple parameters
Source: Nucleic Acids Res. 2015 Apr 16;43(Web Server issue):W7–W14. doi: 10.1093/nar/gkv318 (PMC4489236; doi:10.1093/nar/gkv318)
Supplement: SUPPLEMENTARY DATA [file supp_43_W1_W7__index.html]

GUIDANCE2: accurate detection of unreliable alignment regions accounting for the uncertainty of multiple parameters — GUIDANCE2: accurate detection of unreliable alignment regions accounting for the uncertainty of multiple parameters — SUPPLEMENTARY DATA 

# GUIDANCE2: accurate detection of unreliable alignment regions accounting for the uncertainty of multiple parameters

## SUPPLEMENTARY DATA

**Files in this Data Supplement:**

- SUPPLEMENTARY FIGURES
