# Supplementary material for: GUIDANCE2: accurate detection of unreliable alignment regions accounting for the uncertainty of multiple parameters
Source: Nucleic Acids Res. 2015 Apr 16;43(Web Server issue):W7–W14. doi: 10.1093/nar/gkv318 (PMC4489236; doi:10.1093/nar/gkv318)
Supplement: SUPPLEMENTARY DATA [file supp_gkv318_nar-00479-web-b-2015-File005.pdf]

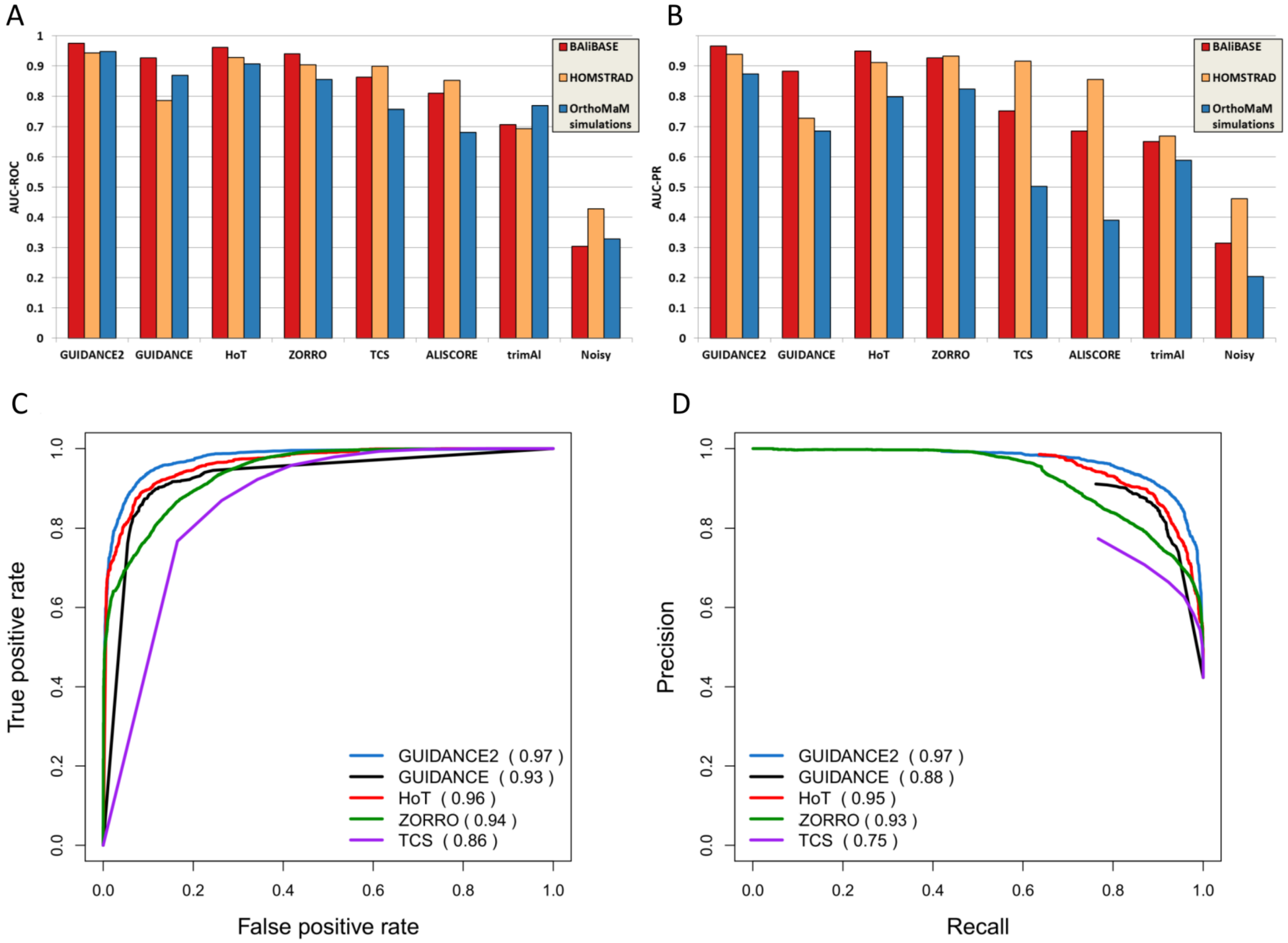

Figure S1: Quantitative comparison of all MSA reliability algorithms for different datasets when using ClustalW to align the sequences. (A) AUC-ROC and (B) AUC-PR. Performance curves of the five leading methodologies over the BAliBASE dataset. (C) ROC and (D) Precision-Recall.

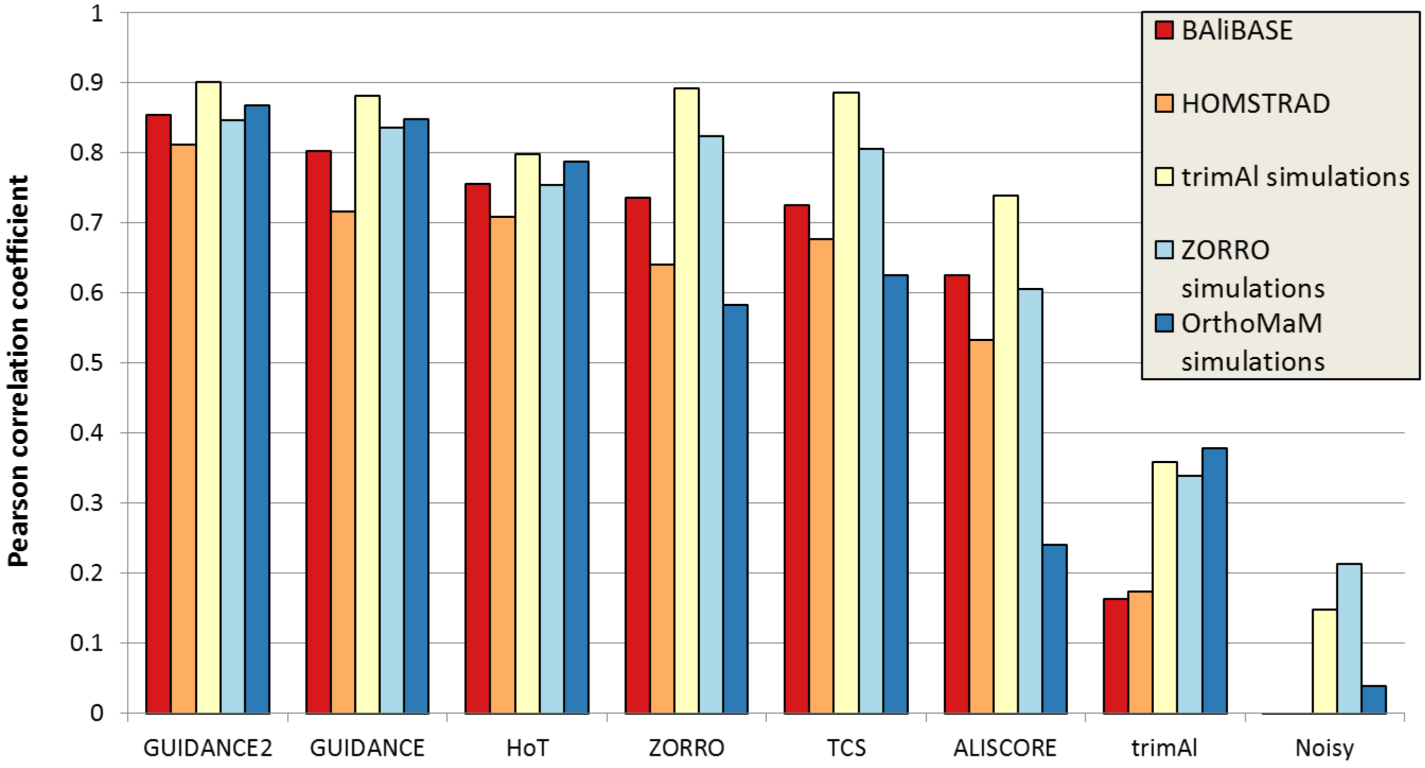

Figure S2: Pearson correlation coefficients between each method's column reliability score and the fraction of correctly aligned pairs in each column for all five datasets. For GUIDANCE2, GUIDANCE and HoT the res_pair_col score (*i.e.*, the SPC score) was used for the correlation analysis.

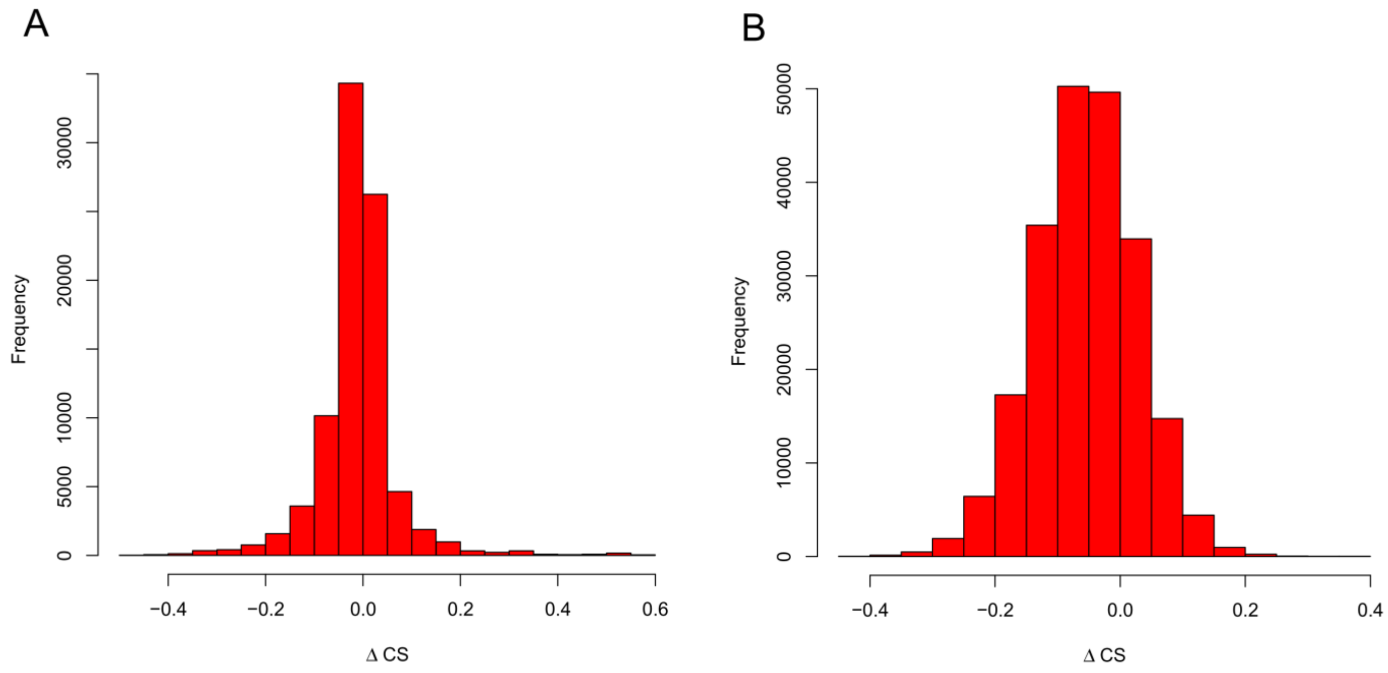

Figure S3: Difference in average CS between alternative and base MSAs, based on the BAliBASE benchmark (A) and OrthoMAM simulated data (B).

	BaliBASE	HOMSTRAD	ZORRO simulations	trimAl simulations	OrthoMaM simulations
Number of sequences in MSA	29 (26)	7 (5)	100 (0)	46 (16)	40 (0)
MSA length	130 (119)	257 (182)	1653 (1308)	1796 (938)	802 (478)
Gap percentage	1.5E-05	0.17 (0.1)	0.72 (0.08)	0.74 (0.11)	0.46 (0.09)
Sequences similarity	0.11 (0.07)	0.19 (0.13)	0.03 (0.02)	0.02 (0.02)	0.38 (0.11)

Table S1: Statistics for all benchmark datasets used to evaluate MSA reliability methodologies performance. Mean value for all dataset is indicated for each dataset, standard deviation is indicated in parentheses. The similarity score was calculated using the trimAl software. For exact score definition see section 1.2.2 in supplementary material of Capella-Gutierrez et al (2009).
